# Orbital glomus tumor in an Asian patient

**DOI:** 10.1186/1471-2415-12-62

**Published:** 2012-12-05

**Authors:** Minwook Chang, Youngseok Lee, Sehyun Baek, Tae Soo Lee

**Affiliations:** 1 Department of Ophthalmology, Korea University College of Medicine, Seoul, Korea; 2 Department of Pathology, Korea University College of Medicine, Seoul, Korea

**Keywords:** Orbital glomus tumor, Asian patients, Recur, Rapid growth, Painless

## Abstract

**Background:**

This report describes a recurrent orbital glomus tumor in an Asian patient.

**Case presentation:**

A healthy 50-year-old Korean man had progressive right exophthalmos and a soft mass on his right lower lid for 6 months. We evaluated the mass using CT and MRI, and performed excisional biopsy and pathologic examination. Pathologically, the mass was a glomus tumor. Although proptosis of the right eye decreased, one month after surgery it increased to almost the same level as before surgery.

**Conclusions:**

This is the first report of an Asian patient with an orbital glomus tumor that demonstrated rapid re-growth after incision without pain or visual problems.

## Background

Glomus tumors are rare, benign neoplasms of the glomus body, a specialized thermoregulatory arteriovenous structure. These tumors most commonly present in the dermis of the digits and palms, [1] and rarely in the orbit. We describe the unique features of a large orbital glomus tumor that developed and re-grew rapidly in an Asian patient.

## Case presentation

**Figure 1:**
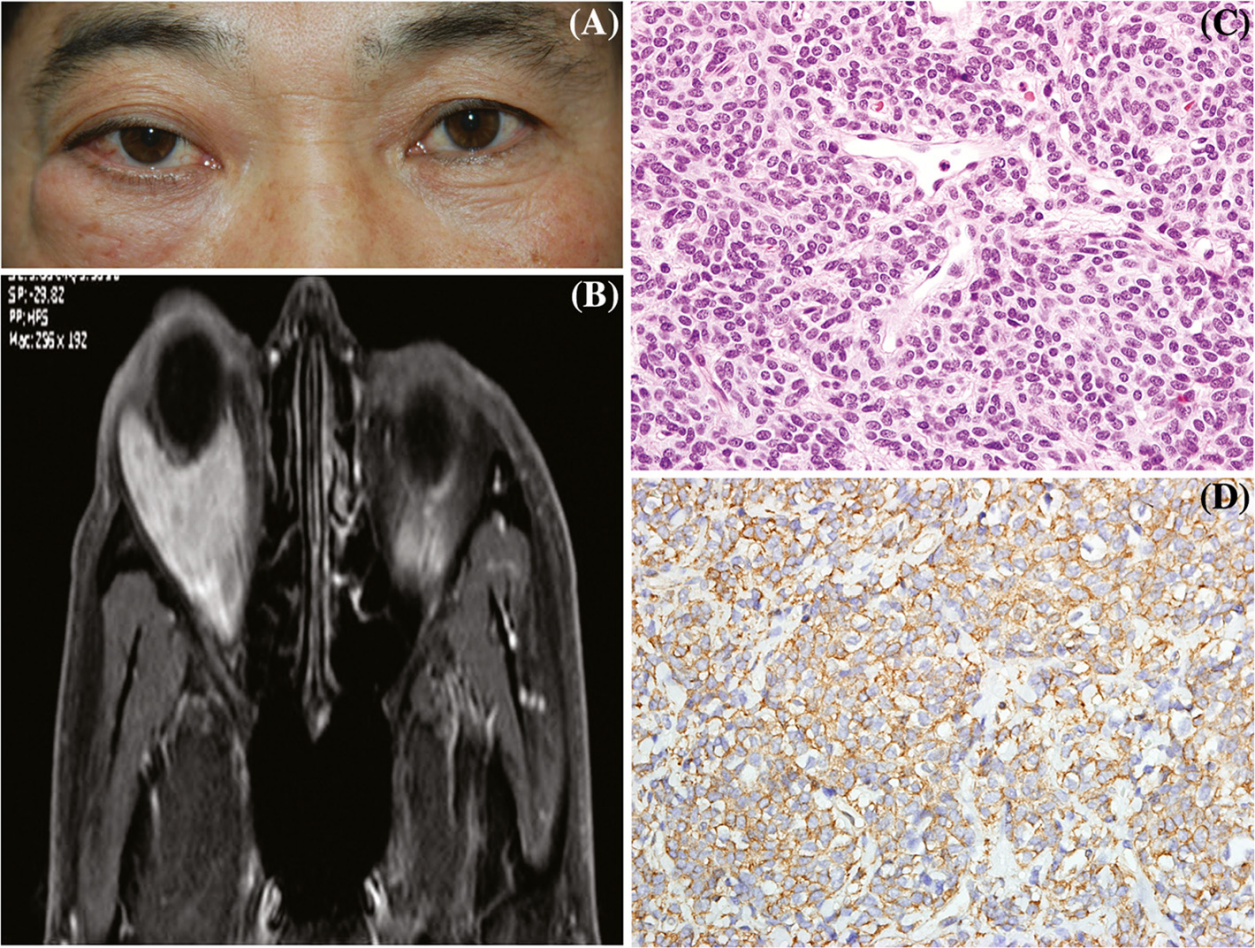
**Photographs demonstrating right lower lid swelling and proptosis of the right eye (A).** T1-weighted magnetic resonance imaging demonstrated a large, lobulated, smooth-margined mass in the lower lateral aspect of the right orbit, measuring 28x33x32mm in volume with a low signal that diffusely enhanced with gadolinium (**B**). Tumor cells were arranged in organoid and sheet-like patterns, and contained intimately associated capillary-sized blood vessels. Individual tumor cells were round, oval, polyhedral, or fusiform. Nuclei were round or ovoid and centrally placed in pale or slightly eosinophilic cytoplasm. Cell borders were not clearly delineated. Tumor cells were intermingled with thin fascicles of bland spindle cells (hematoxylin-eosin, original magnification x400) (**C**) and showed strong cytoplasmic positivity for smooth muscle actin (SMA) (immunoperoxidase/hematoxylin counterstain, original magnification x400) (**D**).

Written informed consent was obtained from the patient for the publication of this case report and any accompanying images. A copy of the written consent is available for review by the Editor-in-Chief of this journal. A healthy 50-year-old Korean man presented with progressive right exophthalmos and a soft mass on his right lower lid that had persisted for 6 months (Figure 1A). He had neither pain nor visual symptoms. On examination, his visual acuity was 20/20 OU. Pupillary, funduscopic, tonometric, and ocular movements were normal. There was 6 mm of right proptosis (Hertel). Although mild chemosis was found on the right eye, no specific pathologic signs were found. On computed tomography (CT), a well-enhanced mass with dimensions of 38x31x26 mm was noted in the right intra- and extra-conal spaces. The mass appeared to be solid with homogeneous consistency. No bone destruction was evident on the CT scan. A magnetic resonance image (MRI) collected for further evaluation showed a large, lobulated, smooth-margined mass in the lower lateral aspect of the right orbit, measuring 28x33x32mm in volume. The largest portion of the mass was located intraconally and focally protruded extraconally in the infero-lateral section. The mass showed a slightly low signal on T1-weighted imaging that was moderately enhanced by contrast study (Figure 1B). The right optic nerve was elevated due to mass effect, and 3/4 of the optic nerve was enclosed within the mass. The clinical diagnosis was a cavernous hemangioma or, less likely, a malignant lymphoma.

**Figure 2:**
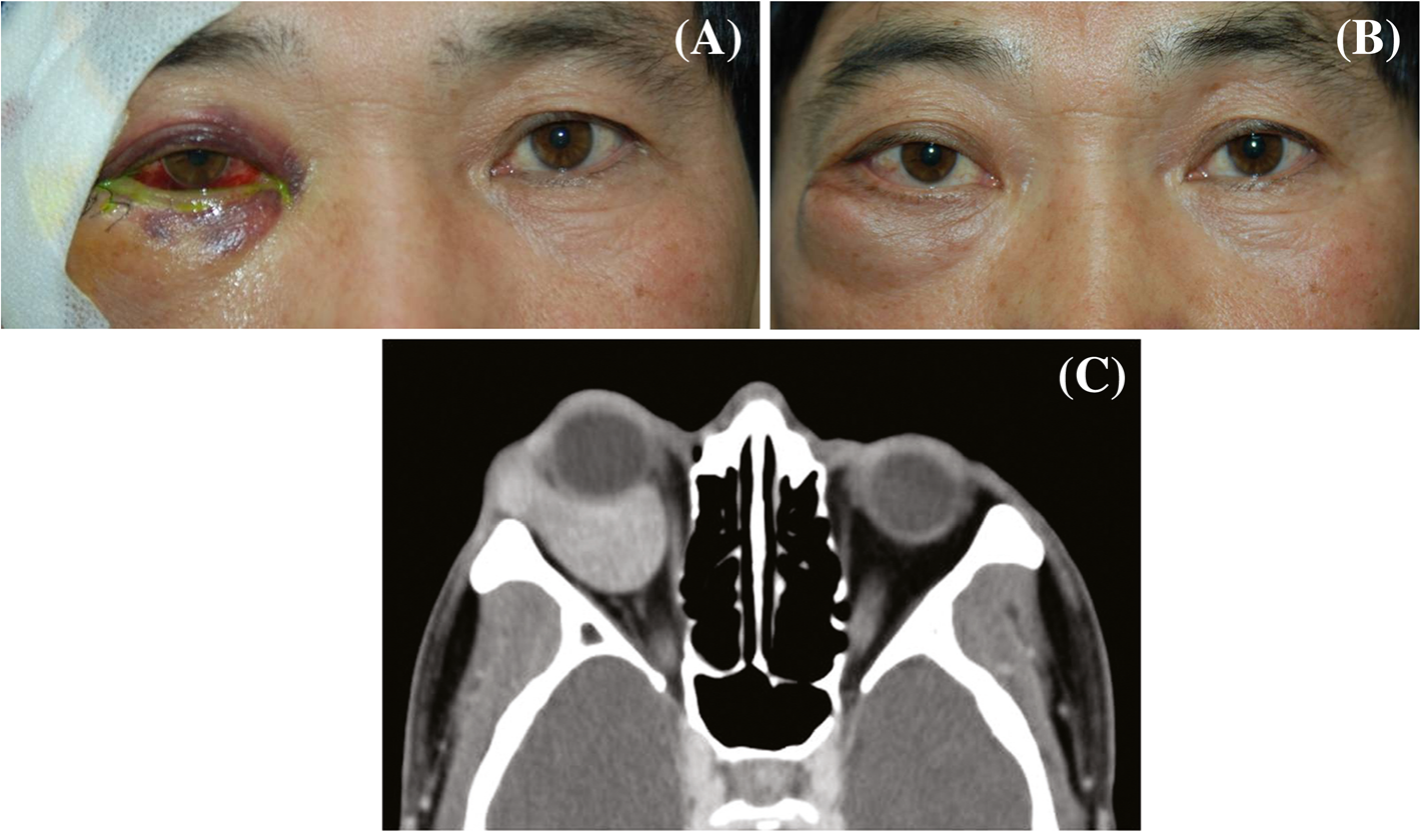
**Photographs taken the next day of surgery demonstrating decrease proptosis and lid swelling (A).** Photographs taken one month after surgery demonstrating recurred right lower lid swelling and proptosis of the right eye (**B**). Follow-up computerized tomography demonstrated that mass size was increased to that of the initial mass (**C**).

To confirm the diagnosis, an incisional biopsy was performed under local anesthesia. A subciliary incision exposed the mass to reveal a smooth, round, highly vascularized tumor. After controlling the extensive bleeding that was encountered, two fragments of the tumor were obtained. The fragments were irregular and multi-lobulated reddish masses measuring 0.9x0.7x0.6cm and 0.6x0.5x0.2cm. Pathologically, tumor cells were arranged in organoid and sheet-like patterns with intimately associated, capillary-sized blood vessels. The individual tumor cells were round, oval, polyhedral, or fusiform. Nuclei were round or ovoid and centrally placed in pale or slightly eosinophilic cytoplasm. The cell borders were not clearly delineated. Tumor cells were intermingled with thin fascicles of bland spindle cells (Figure 1C) and showed strong cytoplasmic positivity for smooth muscle actin (SMA) (Figure 1D). These findings were consistent with the diagnosis of a glomus tumor. After partial excision, proptosis of the right eye decreased to 4 mm, and lower lid swelling visibly reduced (Figure 2A) However, 1 month after surgery, the proptosis increased to 6 mm, which was almost the same level as before surgery. Lower lid swelling had also increased (Figure 2B). A follow-up CT scan indicated that mass size had increased to the same size as the initial mass prior to excision (Figure 2C). Interestingly, the patient still had no specific symptoms. Visual acuity, visual field, and intra-ocular pressure (IOP) were all within normal ranges. Radiotherapy or debulking surgery were recommended to address the possibility of malignancy, but the patient refused any other treatment. Six months after surgery, the patient was stable. However, his proptosis had increased to 7 mm.

## Conclusions

To our knowledge, only 4 other cases of orbital glomus tumors have been reported, and none of them were in Asian patients [2-5]. We reviewed these previous reports and summarized key characteristics to compare to our case (Table 1). The MRI and gross findings of the tumor presented here are very similar to those of the case reported by Pribila et al. [4]. However, the tumor in our study was histopathologically compatible with a glomus tumor proper, while the tumor in the previous study showed both glomus cell tumor and glomangioma characteristics. The previous patient also had pain at presentation, while ours had no pain or vision symptoms. Unlike in the three previous reports, the tumor in our patient grew very rapidly. In addition, the tumor showed rapid re-growth months after surgical debulking. This fast re-growth may have been an atypical glomus tumor, which is defined by Folpe et al. as a glomus tumor with uncertain malignant potential [6]. Fortunately, our patient had no visual problems, but we recommended continued close observation and further evaluation to rule out the possibility of malignancy. To our knowledge, this is the first report in an Asian patient of an orbital glomus tumor that demonstrated rapid asymptomatic re-growth after surgical debulking.

**Table 1:** Characteristics of previously reported orbital glomus tumors

**Report (year)**	**Patient**		**Onset**	**Proptosis**	**Pain**	**Other signs**	**Radiologic findings**	**Management**	**Gross findings**	**Pathology (type)**	**Re-growth**
	**sex**	**age**									
Neufeld et al. (1994)	F	35	1yr	4mm	none	none	2.4x1.4x1.6 cm Solid homogenous (CT)	cryoextraction	Fully encapsulates with a purple-blue color	Glomus cell tumor proper	None
Shields et al. (2006)	M	17	5yrs	none	none	none	-	debulking	Blue-gray subconjunctival lesion involving medial and superior rectus muscles	glomangioma	None
Pribila et al. (2010)	F	19	-	3.5mm	Painful burning sensation	Limited abduction and supraduction	irregular, lobulated, 2.6x3.5x3.3 cm isointense to muscle on T1 weighting imaging, which was diffusely enhanced with gadolinium (MRI)	exenteration	Extensive bleeding and friable	Both glomus cell tumor proper and glomangioma	None
Ulivieri et al. (2012)	F	29	-	-	Painful	none	A well-defined circumscribed mass,displacing the globe and lateral rectus muscle inferotemporally	Excision (Kronlein approach)	-	glomangioma	none
Our report	M	50	6mon	6mm	none	none	smooth marginated, lobulated 2.8x3.3x3.2 cm, located intraconally and extraconally, low signals on T1 weighting imaging, which were moderately enhanced with gadolinium (MRI)	Partial excision	Reddish and pinkish colored multilobulated mass	Glomus cell tumor proper	Complete

## Competing interests

The authors declare that they have no competing interests.

## Authors’ contributions

TSL, MC, YL and SB carried out the case report’s study, participated in the sequence alignment and drafted the manuscript. All authors read and approved the final manuscript.

## Pre-publication history

The pre-publication history for this paper can be accessed here:

http://www.biomedcentral.com/1471-2415/12/62/prepub
